# Case Report: Lymphodepletion followed by CAR-T cell therapy with Idecabtagen vicleucel in a patient with severe renal impairment

**DOI:** 10.3389/fonc.2023.1288764

**Published:** 2023-11-14

**Authors:** Franziska Marquard, Claudia Langebrake, Dietlinde Janson, Maida Mahmud, Adrin Dadkhah, Nicolaus Kröger, Francis Ayuk

**Affiliations:** ^1^Department of Stem Cell Transplantation, University Medical Center Hamburg-Eppendorf, Hamburg, Germany; ^2^Department of Medicine, Hospital Pharmacy, University Medical Centre Hamburg-Eppendorf, Hamburg, Germany; ^3^Department of Medicine (3rd), University Medical Center Hamburg-Eppendorf, Hamburg, Germany

**Keywords:** CAR-T-cell therapy, lymphodepletion, GFR < 30ml/min, fludarabine, cyclophosphamide, dialysis

## Abstract

Acute kidney injury and chronic kidney disease is common in multiple myeloma. Fludarabine which is part of lymphodepletion before CAR-T cell therapy is renally eliminated and its use is not recommended for patients with severe renal impairment defined as a glomerular filtration rate below 30ml/min/1.73m^2^. We administered fludarabine to a 58-year-old female patient with myeloma-associated severe renal impairment as part of lymphodepletion before Idecabtagen vicleucel infusion. Fludarabine was administered in reduced dose (15mg/m^2^) and cyclophosphamide with a dose of 300mg/m^2^ followed by hemodialysis over six hours using a larger filter (FX-100). The therapy was well tolerated with excellent CAR-T cell expansion and complete remission which is ongoing now beyond 12 months.

## Introduction

Acute kidney injury and chronic kidney disease are common consequences of multiple myeloma due to hypercalcemia and light chain disease associated nephropathy (1). At diagnosis, serum creatinine level of 2 mg/dL or higher is seen in approximately 20% of patients (2). As many therapeutic agents are either nephrotoxic, renally eliminated or even both, treatment of myeloma patients with severe kidney disease is challenging. BCMA targeted CAR-T cells Idecabtagen vicleucel (ide-cel or bb2121) are approved for the treatment of relapsed and refractory multiple myeloma, owing to excellent response rates of 73% in patients who had previously received at least three therapy regimens including a proteasome inhibitor, an immunomodulatory agent, and an anti-CD38 antibody. Prior to CAR-T cell infusion, lymphodepletion is performed with fludarabine (30 mg per m^2^ of body-surface area per day) and cyclophosphamide (300 mg per m^2^ per day) (3) over three days to facilitate CAR-T cell engraftment and expansion. The total clearance of the main metabolite 2F-Ara-A in plasma correlates with creatinine clearance, indicating the importance of renal excretion for the elimination of the substance. Increased total exposure (AUC of 2F-Ara-A) has been demonstrated in patients with impaired renal function. Clinical data on patients with renal impairment (creatinine clearance < 70 ml/min) are limited. Taking into account that 60% of the primary metabolite of fludarabine, F-ara-A is eliminated renally (4), dose adjustment for patients with impaired kidney functions are essential to avoid severe toxicities, such as myelosuppression and neurotoxicity. For glomerular filtration rates (GFR) between 70 and 30 ml/min/1.73m^2^, dose reduction is recommended (5). Below 30ml/min/1.73m^2^, the use of fludarabine is contraindicated according to the summary of product characteristics. Still, adequate dosing of lymphodepletion, particularly fludarabine, may be associated with improved CAR-T cell efficacy (6). In this report, we present the case of a patient with severe renal impairment who was successfully treated with fludarabine, and cyclophosphamide followed by ide-cel for relapsed/refractory multiple myeloma.

## Case

A 58-year-old female patient suffering from relapsed and refractory multiple myeloma of kappa light chain type was admitted to our department to undergo CAR-T cell therapy (Abecma, Ide-cel). At initial diagnosis she presented with multiple osteolysis in lumbar spine and pelvis along with 60% of plasma cells in bone marrow biopsy. Cytogenetically her disease was classified as high risk due to 4;14 translocation, R-ISS stage was stage II. As first line therapy she received an induction regimen with cyclophosphamide/bortezomib/dexamethasone followed by high dose melphalan. Thereafter, under lenalidomide maintenance she stayed in complete remission for 2 years and three months until disease progression. The patient then received local radiotherapy of the left iliac bone and daratumumab/Bortezomib/dexamethasone but progressed after only two months. Next, Carfilzomib/Dexamethasone was administered, and the patient stayed in partial remission for three months before further disease progression again, leading to CAR-T cell indication. Due to further progression on bridging therapy with 2 cycles of elotuzumab/pomalidomide/dexamethasone treatment was changed to isatuximab/carfilzomib/dexamethasone and two cycles were administered prior to admission for CAR-T cell therapy. At this time, she presented with acute kidney injury (serum creatinine 2.69mg/dL, CKD-EPI estimated GFR 20ml/min/1.73m^2^) and progressive disease with elevated kappa light chains in blood and urine as well as new osteolytic lesions and new extramedullary manifestations in a CT and MRI scan. Another cycle of isatuximab/carfilzomib/dexamethasone was initiated but failed to achieve a response. Free kappa light chains increased to 15105.56 with a kappa/lambda ratio of 18881.95. Eventually, chemotherapy with cyclophosphamide/etoposide/dexamethasone was initiated. After chemotherapy serum kappa light chains decreased to 8732.90 (kappa/lambda ratio 6237.79) and kidney function improved slightly but GFR stayed below 30ml/min/1.73m^2^. Following quality release of ide-cel we administered fludarabine and cyclophosphamide using the dose adjustment and dialysis strategy previously published in the context of simultaneous allogeneic and kidney transplantation (7). Briefly, fludarabine was reduced to 15mg/m^2^. Cyclophosphamide dose was 300mg/m^2^ daily for three days. Cyclophosphamide was administered at 7 and Fludarabine at 8pm in the evening. The following morning, the patient was dialyzed using a larger filter (FX 100). We decided to apply full dose of cyclophosphamide at least 12h before dialysis. Cyclophosphamide serum concentration is elevated in patients harboring kidney disease but it is partly removed during hemodialysis (8). Lymphodepletion was well tolerated. Three days after the last dose of fludarabine, ide-cel was infused at a dose of 4x 486,1 x 10^6^ cells. The patient developed CRS grade 2 with fever and dyspnea on day 1, which was successfully treated with two doses of tocilizumab. The patient developed hematologic toxicity including Grade IV neutropenia and thrombocytopenia and grade III anemia. Six erythrocyte concentrates and two thrombocyte concentrates were transfused. Neutropenia < 1000µl was present for 14 days beginning at day -1 before CAR-T cell infusion until day 12. Two doses of granulocyte-colony stimulating factor were applied during this phase. Apart from a symptomatic episode of hypocalcemia with tachycardia and prolongation of the QT interval, which was possibly associated with tumor lysis, no other severe complications were observed. The patient was discharged to outpatient care on day 17 after CAR-T cell infusion.

**Figure 1:**
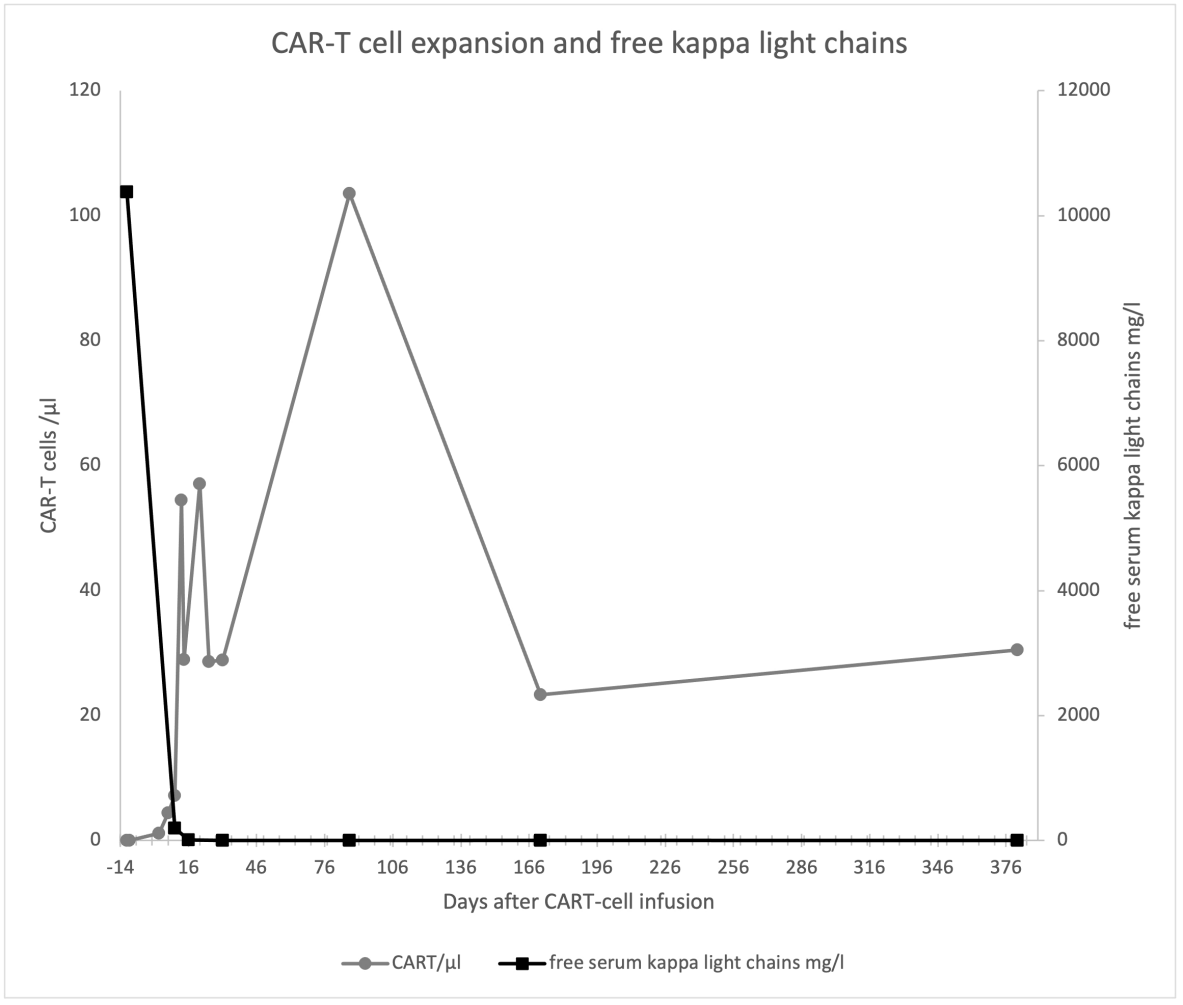
CAR-T cell expansion and free serum kappa light chains.

CAR-T cell expansion was excellent and light chains rapidly decreased to normal range (**Figure 1**). Serum, urine, bone marrow analysis as well as MRI and PET-CT scans 9 months after CAR-T cell therapy revealed complete remission, which was ongoing at last visit one year after treatment.

**Figure 2:**
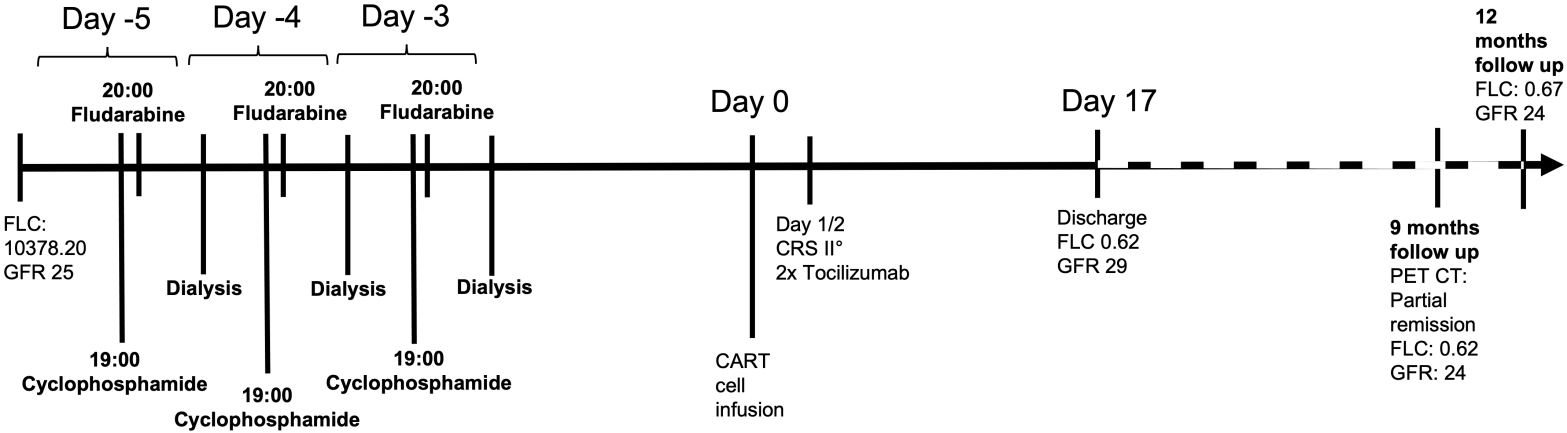
Timeline.

Kidney function has also remained stable with CKD-EPI estimated GFR of 24ml/min at last follow-up. A timeline from the beginning of lymphodepletion can be found in **Figure 2**.

## Discussion and literature review

As shown in **Table 1**, reports on CAR-T cell therapy in patients with eGFR below 30ml/min/1.73m^2^ are very limited. Hunter et al. and Wood et al. each reported 2 patients with end stage kidney disease receiving CD19 targeted CAR-T cells. Hunter et al. reduced fludarabine doses, and hemodialysis was performed 12 hours later as reported by Chen et al. while Wood et. al performed hemodialysis 12h after the first and 12h after the third fludarabine dose (7, 9, 10). CRS and ICANS was seen in three patients. Information on cytopenia was not available. All patients responded to therapy. Very recently, 3 weeks after submission of our report, a retrospective multicentre observational study was published by Sidana et al including 11 patients with GFR < 30ml/min receiving ide-cel for multiple myeloma (11). Administration of lymphodepletion varied between the participating institutes but apparently most of the patients received fludarabine with 25-50% dose reduction without hemodialysis. In this group of patients, cytopenia was not seen more often than in patients without renal impairment. CRS was observed in 100% and ICANS in 40% without significant difference to the group with normal kidney function. The overall response rate and rates of complete remission were also not significantly different than those in patients with a GFR > 30.

**Table 1:** Literature review.

Author	Patients	Disease	Lymphodepletion	Drug	GFR	disease response	CRS/ICANS	cytopenia
Hunter et al., 2022 (9)	n = 2	DLBCL	dose adjusted fludarabine + cyclophosphamide followed by hemodialysis 12h after application	1: axicabtagene ciloleucel 2: lisocabtagene maraleucel	hemodialysis required	1: complete remission at 9 months follow up 2: complete remission, cns relapse 4 months after CAR-T infusion	1: - 2: CRS I°, ICANS II°	not available
Wood et al., 2022 (10)	n = 2	1: DLBCL 2: MCL	dose adjusted fludarabine + cyclophosphamide followed by hemodialysis 12h after the first and third application	1: tisagenlecleucel 2: brexucabtagene autoleucel	hemodialysis required	1: complete remission on day 30, relapse on day 271 (lungs, liver, bones, and musculature) 2: partial remission on day 30, relapse on day 90 after CAR-T infusion	1: CRS I° ICANS IV° 2: CRS II°	not available
Sidana et al., 2023 (11)	n = 11	Multiple myeloma	variations depending on institutional protocols: 25-50% reduction of fludarabine + cyclophosphamide, no hemodialysis if not dialysis dependent	idecabtagen vicleucel	<30ml/min, one patient required hemodialysis	GFR<50 ml/min: (n=28): - day 30 ORR 92%, 28% >/= CR - after three months ORR 70%, 41% >/= CR	CRS: 100%, 9,1%> grade III or higher ICANS: 40%, 20% grade III or higher	Day 60: - 22% anemia grade 3 or higher - 11% neutropenia grade.3 or higher - 56% thrombocytopenia grade 3 or higher

DLBCL, diffuse large b-cell lymphoma; MCL, mantle-cell lymphoma; CRS, cytokine release syndrome; ICANS, immune effector cell-associated neurotoxicity syndrome.

CAR-T cells are a novel and very potent treatment option for patients with relapsed and refractory multiple myeloma and non-Hodgkin lymphoma. As impaired kidney function is a common problem especially in multiple myeloma, it is important not to exclude this large group of patients from these emerging new therapeutic options. Lymphodepletion has an important impact on progression free survival in CD19-targeted CAR-T cells in non-Hodgkin Lymphoma (6), so dosing modifications or finding alternative agents that may help circumvent current limitations is of utmost interest. Our patient showed excellent CAR-T cell expansion and has stayed in complete remission now lasting more than 12 months.

Chen et al. showed promising results in their study on five patients receiving haploidentical hematopoietic stem cell transplantation after conditioning with fludarabine, total body irradiation and cyclophosphamide. The patients received hemodialysis between 6 and 12 hours after fludarabine administration. While three of them received longer dialysis over six hours with a larger dialyzer (FX 100), two patients received standard dialysis. One of the patients died from fludarabine neurotoxicity, so the investigators decided to perform fludarabine pharmacokinetic analysis, which did not show any difference to patients receiving fludarabine with normal kidney function (7).

Hematological toxicity was present in our patient with grade IV neutropenia and thrombocytopenia and grade III anemia. In patients with normal kidney function grade III or IV neutropenia was observed in 89%, anemia in 60%, and thrombocytopenia in 52% (3).

In our patient, grade III thrombocytopenia was still present on day 60 after CAR-T infusion, while the patient did not need transfusion or granulocyte-colony stimulating factor anymore. In Sidana et al., >/= grade III thrombocytopenia was observed in 73% in the group of patients with GFR < 30 ml/min on day 60 after CAR-T infusion, while >/= grade III anemia and neutropenia were still present in 36%. On day 90 after CAR-T infusion, Sidana et al. still observed >/= grade III anemia and thrombocytopenia in 10 and 30%, respectively, while our patient no longer demonstrated grade III cytopenias (11).

In future studies, it would be useful to do fludarabine pharmacokinetics and, to determine the optimal target exposition of fludarabine in terms of toxicity and efficacy.

## Conclusion

CAR-T cell therapy is an important therapy option for patients with relapsed and refractory multiple myeloma and should also be offered to patients with severe renal impairment and eGFR below 30ml/min/1.73m^2^. For the first time, we performed lymphodepletion with fludarabine and cyclophosphamide followed by long hemodialysis over 6 hours using a larger filter (FX 100). The patient showed excellent CAR-T cell expansion and complete remission of her disease for at least 12 months without severe toxicity events. Further investigations addressing pharmacokinetics of fludarabine under hemodialysis and examining therapy response and toxicity in a larger cohort of patients should be initiated.

## Data availability statement

The original contributions presented in the study are included in the article/supplementary material. Further inquiries can be directed to the corresponding author.

## Ethics statement

Ethical approval was not required for the study involving humans in accordance with the local legislation and institutional requirements. Written informed consent to participate in this study was not required from the participants or the participants' legal guardians/next of kin in accordance with the national legislation and the institutional requirements. Written informed consent was obtained from the individual(s) for the publication of any potentially identifiable images or data included in this article.

## Author contributions

FM: Conceptualization, Writing – original draft, Writing – review & editing. CL: Writing – review & editing. DJ: Writing – review & editing. MM: Writing – review & editing. AD: Writing – review & editing. NK: Supervision, Writing – review & editing. FA: Conceptualization, Writing – original draft, Writing – review & editing. Written informed consent was obtained from the patient in accordance with approval of the local ethics committee (PV7081).
